# Supplementary material for: Physical Activity and Glycemic Control Status in Chinese Patients with Type 2 Diabetes: A Secondary Analysis of a Randomized Controlled Trial
Source: Int J Environ Res Public Health. 2021 Apr 18;18(8):4292. doi: 10.3390/ijerph18084292 (PMC8073010; doi:10.3390/ijerph18084292)
Supplement: Supplementary file 1 [file ijerph-18-04292-s001.zip › ijerph-1143210-supplementary-final.pdf]

International Journal of
Environmental Research and Public Health

MDPI

Table S1. Baseline characteristics of the study participants by intervention status.

Characteristics at Baseline	All Subjects (N = 799)	Intervention Groups			Control Arm (N = 200)	*P-Values* [c]
		Health Literacy (N = 200)	Exercise (N = 200)	Comprehensive (N = 199)		
**Cluster**	8	2	2	2	2	
**Clinic sites**	35	9	9	9	8	
**Age (years)** [a]	66 (59, 71)	67 (60, 71)	66 (60, 72)	66 (59, 72)	65 (59, 69)	*0.06*
**Male** [b]	45.2	44.5	48.0	45.2	43.0	*0.79*
**Educational level** [b]						*<0.001*
Primary school or below	21.7	19.6	16.0	32.7	18.5	
Junior high school	38.2	39.2	33.5	39.7	40.5	
Senior high school	26.2	29.1	30.5	20.1	25.0	
College and above	13.9	12.1	20.0	7.5	16.0	
**Monthly income *per capita* (USD)** [b]						*<0.001*
<308	14.6	16.6	6.1	21.1	14.6	
308-769	58.0	58.3	54.5	59.8	59.6	
>769	27.4	25.1	39.4	19.1	25.8	
**Tobacco smoking** [b]	15.7	14.5	12.1	16.5	19.6	*0.20*
**Alcohol drinking** [b]	11.0	11.7	9.3	8.9	14.3	*0.29*
**Years of diabetes** [a]	9.7 (5.1, 15.1)	10.4 (6.0, 16.1)	9.5 (4.8, 15.9)	9.6 (5.1, 14.6)	9.5 (5.2, 14.6)	*0.31*
**Medication use** [b]						*0.005*
Diabetes pills only	63.8	71.2	64.2	58.6	61.5	
Insulin shot only	8.6	5.8	6.9	8.7	12.8	
Both	21.7	17.8	18.7	28.6	21.6	
Neither	5.9	5.2	10.2	4.1	4.1	
**HbA1c level** [a]	8.1 (7.5, 9.1)	8.1 (7.5, 9.3)	8.0 (7.5, 9.1)	8.1(7.7, 9.0)	8.2(7.5, 9.1)	*0.69*
**Health literacy** [a]	116 (108, 120)	116 (104, 120)	116 (104, 120)	113(97, 120)	116(113, 120)	*<0.001*
**Numeracy skill** [a]	80 (60, 100)	80 (80, 100)	80 (80, 100)	80(60, 100)	100(80, 100)	*<0.001*

[a] Continuous variables presented as median and interquartile range (IQR); [b] Categorical variables presented as percentage; [c] One-way analysis of variance (ANOVA) or Kruskal-Wallis tests for comparisons of continuous variables, chi-square tests for comparisons of categorical variables.

Table S2. Baseline intensity of exercise by HbA1c levels in the study participants.

Type of PA	Men				Women			
	**HbA1c: <7.5% (N = 62)**	**HbA1c: 7.5-8.9% (N = 191)**	**HbA1c: ≥ 9.0% (N = 99)**	***P-Values*** [c]	**HbA1c: <7.5% (N= 104)**	**HbA1c: 7.5-8.9% (N = 213)**	**HbA1c: ≥ 9.0% (N = 115)**	***P-values*** [c]
**Exercise**								
Participation [a]	51.6	48.7	58.6	*0.28*	55.8	45.5	41.7	*0.10*
Intensity (MET-h/w) [b]								
Moderate	0 (0, 16.0)	0 (0, 12.0)	3.3 (0, 18.7)	*0.15*	0 (0, 12.0)	0 (0, 12.0)	0 (0, 8.0)	*0.57*
Vigorous	0 (0, 0)	0 (0, 0)	0 (0, 0)	*0.61*	0 (0, 0)	0 (0, 0)	0 (0, 0)	*0.42*
Total	3.3 (0, 28.0)	0 (0, 24.0)	10.3 (0, 28.0)	*0.26*	6.7 (0, 24.0)	0 (0, 18.7)	0 (0, 20.0)	*0.18*
**Commuting activities**								
Participation [a]	80.7	79.6	77.6	*0.62*	78.9	68.1	69.6	*0.13*
Intensity (MET-h/w) [b]								
Cycling	0 (0, 2.0)	0 (0, 4.0)	0 (0, 0)	*0.91*	0 (0, 0)	0 (0, 0)	0 (0, 0)	*0.27*
Walking	22.0 (6.0, 28.0)	14.0 (4.0, 28.0)	14.0 (5.3, 28.0)	*0.20*	20 (6.0, 28.0)	14.0 (0, 28.0)	14.0 (0, 28.0)	*0.07*
Total	27.0 (8.0, 37.3)	18.0 (4.0, 28.0)	20.0 (10, 41.3)	*0.26*	28 (6.7, 30.3)	14.0 (0, 28)	14.0 (0, 28.0)	*0.02*
**Housework activities**								
Participation [a]	82.3	82.2	78.8	*0.53*	97.1	92.5	94.8	*0.24*
Intensity (MET-h/w) [b]	21.0 (9.0, 42.0)	15.0 (4.0, 31.5)	15.0 (4.5, 30.0)	*0.17*	42.0 (21, 52.5)	42.0 (21.0, 63.0)	37.5 (21, 45.5)	*0.53*
**Sedentariness** [b]								
MET/h-w	28.0 (21.0, 42.0)	35.0 (21.0, 42.0)	29.2(21.0, 42.0)	*0.85*	28.0 (17.5, 37.3)	28.0 (18.7, 42.0)	28.0 (21.0, 38.5)	*0.77*

[a] Continuous variables presented as median and interquartile range (IQR); [b] Categorical variables presented as percentage; [c] One-way analysis of variance (ANOVA) or Kruskal-Wallis tests for comparisons of continuous variables, chi-square tests for comparisons of categorical variables.

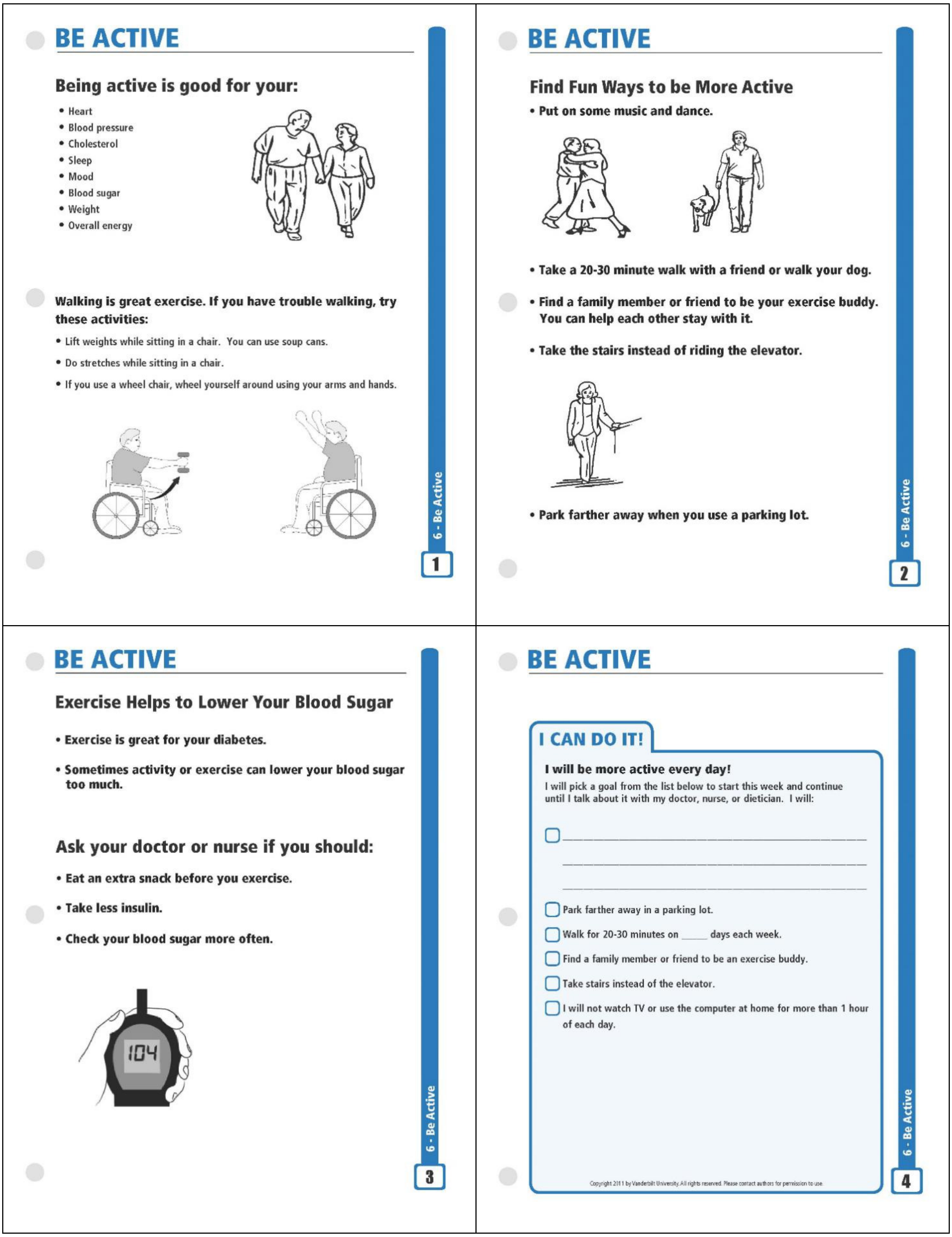

BE ACTIVE

Being active is good for your:

- Heart
- Blood pressure
- Cholesterol
- Sleep
- Mood
- Blood sugar
- Weight
- Overall energy

Walking is great exercise. If you have trouble walking, try these activities:

- Lift weights while sitting in a chair. You can use soup cans.
- Do stretches while sitting in a chair.
- If you use a wheel chair, wheel yourself around using your arms and hands.

6 - Be Active

1

BE ACTIVE

Find Fun Ways to be More Active

- Put on some music and dance.
- Take a 20-30 minute walk with a friend or walk your dog.
- Find a family member or friend to be your exercise buddy. You can help each other stay with it.
- Take the stairs instead of riding the elevator.
- Park farther away when you use a parking lot.

6 - Be Active

2

BE ACTIVE

Exercise Helps to Lower Your Blood Sugar

- Exercise is great for your diabetes.
- Sometimes activity or exercise can lower your blood sugar too much.

Ask your doctor or nurse if you should:

- Eat an extra snack before you exercise.
- Take less insulin.
- Check your blood sugar more often.

104

6 - Be Active

3

BE ACTIVE

I CAN DO IT!

I will be more active every day!

I will pick a goal from the list below to start this week and continue until I talk about it with my doctor, nurse, or dietician. I will:

- ☐ ______________________________
  ____
  ____
- ☐ Park farther away in a parking lot.
- ☐ Walk for 20-30 minutes on _____ days each week.
- ☐ Find a family member or friend to be an exercise buddy.
- ☐ Take stairs instead of the elevator.
- ☐ I will not watch TV or use the computer at home for more than 1 hour of each day.

Copyright 2011 by Vanderbilt University. All rights reserved. Please contact authors for permission to use.

6 - Be Active

4

Figure S1. Exercise module in the PRIDE toolkit
